# Proteolysis at the Archaeal Membrane: Advances on the Biological Function and Natural Targets of Membrane-Localized Proteases in *Haloferax volcanii*

**DOI:** 10.3389/fmicb.2022.940865

**Published:** 2022-06-24

**Authors:** Rosana E. De Castro, María I. Giménez, Micaela Cerletti, Roberto A. Paggi, Mariana I. Costa

**Affiliations:** Instituto de Investigaciones Biológicas (IIB-CONICET-UNMDP), Universidad Nacional de Mar del Plata, Mar del Plata, Argentina

**Keywords:** archaea, *Haloferax volcanii*, membrane proteases, protease targets, archaeal physiology, proteolysis

## Abstract

Proteolysis plays a fundamental role in many processes that occur within the cellular membrane including protein quality control, protein export, cell signaling, biogenesis of the cell envelope among others. *Archaea* are a distinct and physiologically diverse group of prokaryotes found in all kinds of habitats, from the human and plant microbiomes to those with extreme salt concentration, pH and/or temperatures. Thus, these organisms provide an excellent opportunity to extend our current understanding on the biological functions that proteases exert in cell physiology including the adaptation to hostile environments. This revision describes the advances that were made on archaeal membrane proteases with regard to their biological function and potential natural targets focusing on the model haloarchaeon *Haloferax volcanii.*

## Introduction

Proteolysis occurring at the cell surface is necessary to maintain membrane protein homeostasis, protein export, cell signaling and biogenesis of the cell envelope among other membrane-related processes. The targets of these enzymes are mainly membrane-anchored and secretory proteins including receptors, transporters and a variety of enzymes such as transferases, oxidoreductases, and hydrolases. Archaeal genomes encode many of the membrane protease families found in bacteria and eukaryotes, however, only a limited number of these enzymes have been studied and their biological function and natural targets in most cases remain unknown. The repertory of membrane-localized proteases that occur in archaeal model organisms as well as their biochemical features were previously reviewed (Maupin-Furlow et al., 2005; De Castro et al., 2006; Rotanova et al., 2006; Ng et al., 2007; Gimenez et al., 2015; Maupin-Furlow, 2018; Pohlschroder et al., 2018).

This article describes advances on the biological role and natural targets of membrane-localized proteases of archaea, taking as case study the model euryarchaeon *Haloferax volcanii*. In recent years, the impact of LonB and Rhomboid RhoII proteases on *H. volcanii* physiology was explored by genetic, physiology as well as different proteomics strategies (Cerletti et al., 2014, 2015, 2018; Costa et al., 2018). Several potential natural targets were identified which allowed to begin unraveling the cellular processes regulated by these proteases. A novel mechanism was addressed in *H. volcanii* which anchors cell envelope proteins (S-layer glycoprotein, SLG) by means of lipid attachment on the C-terminus after processing by the membrane protease archaeosortase A (ArtA) (Abdul Halim et al., 2013). Recently, additional components of the ArtA-dependent cell wall maturation mechanism were discovered, advancing the understanding on the cell envelope biogenesis in archaea.

Table 1 shows a list of membrane-localized proteases of *H. volcanii*, including potential target proteins and the associated biological processes.

**TABLE 1:** Membrane-localized proteases characterized in the haloarchaeon *Haloferax volcanii*.

Name	MEROPS family	Description	Homologs	Locus tag	Protein ID	Active site localization	Biological process/Identified targets	References
**Experimentally characterized proteases**								
LonB	S16	Compartmentalized ATP-dependent protease. Archaeal-type LonB is anchored to cell membrane through a transmembrane segment (TMS) within the AAA+ module. The archaeal LonB has been characterized biochemically and/or at the structural level in various archaea. In eukaryotes and bacteria is involved in protein quality control and regulatory circuits	HvLonB	HVO_0783	D4GTT9	Cytosol	Essential for viability. Carotenoid biosynthesis; Cell shape determination; protein glycosylation/Phytoene synthase, CetZ1, HVO_0704, AglG, Agl10	Maupin-Furlow et al., 2005; Cerletti et al., 2014, 2015, 2018; Ferrari et al., 2020
Archaeosortase	C106	Sortases are membrane-anchored cysteine-transpeptidases (catalytic triad Cys-His-Arg) found in Gram positive bacteria. Process and covalently attach proteins to the cell wall and assemble pilli to the microbial surface. Archaeosortase A is a polytopic transpeptidase which has the conserved active site residues of sortases but unrelated in sequence homology	ArtA	HVO_0915	D4GUZ4	Extracellular	Maturation of SLG (C-terminal processing of SLG)/SLG, HVO_0405	Haft et al., 2012; Abdul Halim et al., 2013, 2015, 2017, 2018; Abdul-Halim et al., 2020
Signal peptidase I (SPI)	S26	Cleaves type I signal peptide of the majority of preproteins transported through the general secretion pathway (Sec) and, most likely, preproteins transported *via* the Twin Arginine Translocation (Tat) pathway as well.	Sec11a Sec11b	HVO_2603 HVO_0002	D4GUC5 L9UJ60	Extracellular	Sec11b is essential for viability. Protein translocation/Sec and (most likely) Tat pre-proteins	Fine et al., 2006; Fink-Lavi and Eichler, 2008
TFPP-like (SPIII)	A24B	Type IV prepilin peptidase (TFPP) cleaves the signal peptide of prepilins and prearchaelins (class III signal peptides) that contain a prepilin processing site preceding the hydrophobic stretch of the signal peptide	PibD	HVO_2993	D4GY85	Intramembrane (I-CliP)	Maturation of components of the pili and archaellum/Pilins 1-6, FlgA1, FlgA2, HVO_2451. HVO_A0632	Tripepi et al., 2010; Esquivel et al., 2013
Rhomboid	S54	Regulatory proteases conserved among the three domains of life. Enzymes with catalytic dyad Ser- His at TMS 4 and 6. The biological relevance of rhomboids in prokaryotes is scarcely known, include control of quorum sensing, and membrane proteostasis	RhoII	HVO_0727	D4GT94	Intramembrane (I-CliP)	SLG N-Glycosylation; cell adhesion; motility/putative substrates PibD, Agl15	Stevenson et al., 2007; Parente et al., 2014; Began et al., 2020; Costa et al., 2020; Liu et al., 2020
**Proteases detected by proteomics**								
HtpX	M48	HtpX is a Zn^2+^-dependent protease, occurs in bacteria and archaea. Protein structure containing an N-terminal domain spanning the cell membrane and a C-terminal proteolytic domain facing the cytoplasm. In bacteria is implicated in the degradation of misfolded proteins generated in the cell envelope under stress conditions	HtpX-like protease 1 HtpX-like protease 2 HtpX-like protease 3	HVO_0102 HVO_2904 HVO_A0045	D4GYU8 L9UHV3 L9UV93	Cytosol	Unknown	Vickerman et al., 2002; Surmann et al., 2016; Schulze et al., 2020
S1P (site -1- protease)	M82	Protease required for the site-1 cleavage of anti-σ factor σ*^W^* in *Bacillus subtilis.* PrsW is the founding member of a family of membrane-embedded metalloproteases with predicted structural similarities to the prenyl endopeptidase Rce1 of *Saccharomyces cerevisiae*	PrsW-like	HVO_0408	D4GRU9	Extracellular	Unknown	Heinrich et al., 2009; Costa et al., 2018; Schulze et al., 2020
CAAX prenyl proteases	M48	Process C-terminal CAAX to localize the proteins into the membrane; involves cysteine prenylation, -AXX cleavage and methylation of the prenylated Cys. In eukaryotes Ras and a-factor converting enzyme 1 (Rce1) is involved in signaling pathways related to cell proliferation, differentiation and carcinogenesis. Rce1 belong to the ABI (Abortive Infection) family of putative integral membrane proteases that occur in all domains of life. CPBP (CAAX proteases and bacteriocin processing enzymes) are present in bacteria and archaea	Abi/CPBP family glutamic-type protease CPBP family glutamic-type protease CPBP family metalloprotease CPBP family metalloprotease CPBP family metalloprotease	HVO_0784 HVO_0737 HVO_0160 HVO_1966 HVO_1997	D4GU09 D4GTA9 D4GZA4 D4GTJ5 D4GTM5	Intramembrane (I-CliP)	Not essential for viability. No growth defects observed in null mutant Unknown	Pei and Grishin, 2001; Manolaridis et al., 2013; Cerletti et al., 2014; Schulze et al., 2020
Aspartyl proteases (GxGD)	A22	Intramembrane aspartyl proteases glycine-x-glycine-aspartate (GxGD) active site motif. Signal Peptide Peptidases (SPPs) degrade signal peptides after protein secretion. SPP and SPP-like proteases participate in regulated intramembrane proteolysis (RIP), protein ectodomain shedding and cleavage of tailed anchored proteins Other members, Presenilin, cleaves β-amyloid precursor protein linked to Alzheimer’s disease. Presenilin homolog (PSH) from archaeon *Methanoculleus marisnigri* provided insight on structure and catalytic mechanism	SPP	HVO_1107	D4GW37	Intramembrane (I-CliP)	Unknown	Li et al., 2013; Mentrup et al., 2020; Schulze et al., 2020; Raut et al., 2021
Rhomboid	S54	See Rhomboid section above	RhoI	HVO_1474	D4GYA9	Intramembrane (I-CliP)	Unknown	Parente et al., 2014; Schulze et al., 2020
S2P (site- 2- protease)	M50	Zn^2+^ - dependent proteases with variable domain architecture. Involved in signaling cascades regulated through sequential intramembrane proteolysis. Occur in all domains of life. Control sporulation, pheromone production, virulence, stress response, lipid biosynthesis. X-ray crystal structures of archaeal S2Ps shed light on catalytic and regulatory mechanisms of S2Ps	M50 family metalloprotease M50 family metalloprotease M50 family metalloprotease M50 family metalloprotease	HVO_0285 HVO_1555 HVO_1870 HVO_1862	D4GZR5 D4GYY4 D4GSX3 D4GSW5	Intramembrane (I-CliP)	Unknown	Feng et al., 2007; Rawson, 2013; Schacherl et al., 2017; Schulze et al., 2020

*Proteases studied at the biochemical, functional and/or proteomic levels were included in the upper section of the table. The lower section shows proteins assigned as membrane protease homologs only detected by proteomics, for which no biological role and/or substrate are known in H. volcanii. Family is provided according to MEROPS: the Peptidase Database (https://www.ebi.ac.uk/merops/) and protein ID according to UniProt database (https://www.uniprot.org).*

## Cellular Processes Regulated by Membrane Proteases in *Haloferax* volcanii

### Carotenoid Biosynthesis

The carotenoid biosynthesis pathway is conserved between photosynthetic organisms and haloarchaea (Rodrigo-Baños et al., 2015). The key step of the pathway is the conversion of two molecules of geranylgeranyl diphosphate (GGPP) into phytoene and is catalyzed by the enzyme phytoene synthase (PSY). In haloarchaea this route leads to the formation of two main products: retinal (C_20_) and the bacterioruberin-type carotenoids (C_50_). Bacterioruberin inserts into the membrane and is responsible for the reddish pigmentation of haloarchaeal cells. It prevents oxidative damage, increases membrane rigidity in hyposaline conditions and provides structural support to archaerhodopsin, a retinal-containing protein complex that is found in some haloarchaea including *Halorubrum* sp. and *Halobacterium* sp. (Giani et al., 2019). The carotenoid biosynthesis pathway and its regulation has been studied in detail at the transcriptional level in photosynthetic organisms (Toledo-Ortiz et al., 2010; Stanley and Yuan, 2019) and to a very limited extent in archaea (Tarasov et al., 2008; Peck et al., 2017, 2019). In the last years post-translational control of PSY by proteolysis emerged as a novel strategy to modulate carotenogenesis in plants (Welsch et al., 2018; Wang et al., 2020). Interestingly, LonB was found to be a negative regulator of carotenoids biosynthesis in the archaeon *H. volcanii* by controlling the turnover (degradation) of PSY (Cerletti et al., 2018).

In *H. volcanii* the membrane-associated LonB is an essential protease meaning that it has a central role in its physiology (Cerletti et al., 2014). *H. volcanii* mutant cells producing suboptimal (reduced) levels of LonB (strain HVLON3) incremented their pigmentation remarkably (Cerletti et al., 2014) and in contrast, when overexpressing LonB, the cells became completely colorless (Sastre et al., 2010; Cerletti et al., 2014). A shotgun proteomics analysis showed that the abundance of PSY was incremented by about 50-fold in HVLON3 compared to the parental strain H26 (Cerletti et al., 2015). Further studies aiming to explore *H. volcanii* proteome turnover under varying LonB concentrations demonstrated that the degradation of PSY was strongly LonB-dependent (Cerletti et al., 2018). Altogether these results indicate that PSY is a (direct or indirect) target of this protease. These studies also show that proteolysis of PSY is a posttranslational mechanism for the control of carotenogenesis that is conserved in distantly related organisms such as plants and archaea.

### Cell Shape and Cell Division

*Haloferax volcanii* cells grown in liquid cultures show an irregular plate-shaped morphology but they differentiate into rods to attain efficient swimming motility. This change in morphology is directed by the tubulin-like protein CetZ1 (HVO_2204) (Duggin et al., 2015). Interestingly, *H. volcanii* mutant cells containing reduced LonB amounts growing in liquid cultures showed unusually irregular-elongated cells compared to the parental strain H26 (Cerletti et al., 2014) while swarmer cells induced to overexpress LonB evidenced a rounded-shaped morphology (Ferrari et al., 2020). Consistent with these phenotypes, high resolution proteomics showed that the abundance and/or turnover of several tubulin homologues were modified depending on LonB expression (Cerletti et al., 2015, 2018). Specifically, the archaeal rod-shape determinant CetZ1 was degraded faster when LonB expression was incremented, which was substantiated by an *in vivo* degradation assay (Ferrari et al., 2020). Under reduced LonB levels CetZ1 was still degraded suggesting that the turnover of this protein may rely on the activity of other protease/s in addition to LonB. *In vivo* crosslinking assays coupled to immunoprecipitation with anti-LonB antibodies identified CetZ1 (and CetZ5) among the co-precipitated LonB partners (Ferrari et al., 2020). Altogether, these results provide evidence that LonB is likely involved in the modulation of *H. volcanii* cell shape by controlling the stability of CetZ1.

As in bacteria, many archaea (including haloarchaea) use the tubulin family proteins FtsZ1 and FtsZ2 to build up the septum during cell division (Liao et al., 2021). However, the regulatory factors affecting this process are largely unknown in these organisms. The Lon protease is a regulator of cell division in bacteria. Upon DNA damage, the inhibitor protein SulA prevents FtsZ polymerization arresting cell division. Once DNA has been repaired, SulA is degraded by the Lon protease allowing FtsZ polymerization. *Escherichia coli* Δ*lon* cells form filaments after irradiation with UV-B light as they cannot complete cell division. A recent work showed that *E. coli* Δ*lon* cells complemented with the recombinant LonB protease from the haloarchaeon *Natrialba magadii* produced shorter filaments after irradiation with UV-B light compared to the control (non-induced cells), and FtsZ1 and FtsZ2 were identified as potential interacting partners with LonB in a pull-down assay (Ferrari et al., 2020). Although SulA homologs have not been identified in archaeal genomes, this preliminary evidence may guide future investigations aiming to explore the implication of LonB in (halo)archaeal cell division.

### Biogenesis of Cell Envelope and Surface Structures

#### Protein Translocation and Secretion

In archaea proteins are transported into or across membranes by means of the General Secretion (Sec) pathway (unfolded substrates), or through the Twin Arginine Translocation (Tat) system (folded proteins) (Pohlschröder et al., 2005; Calo and Eichler, 2011). Even though in most organisms the Tat pathway is used to transport a minor set of proteins, in haloarchaea it is used for the majority of the secretome (Bolhuis, 2002; Rose et al., 2002). Some known Tat substrates are halolysins Nep from *N. magadii* (Ruiz et al., 2012) and SptA from *Natrinema* sp. (Du et al., 2015). Signal Peptidase I (SPI) cleaves the type I signal peptide of the majority of preproteins transported through these pathways (Paetzel et al., 2002; Pohlschröder et al., 2005; Sargent et al., 2006). *Haloferax volcanii* has two SPIs with catalytic activity, Sec11a (HVO_2603) and Sec11b (HVO_0002) (Fine et al., 2006), from which only Sec11b is essential for *H. volcanii* viability.

In *Archaea*, the assembly and structure of the archaellum are similar to that of bacterial type IV pilins (Chaudhury et al., 2018). The preproteins composing these appendages are processed by a specific SP, the type IV prepilin peptidase (TFPP). Haloarchaeal genomes encode a single TFPP and contain several pilin-like genes (Szabó et al., 2007). In *H. volcanii* the TFPP homolog PibD (HVO_2993) is responsible for processing two archaellins and six pilins (Tripepi et al., 2010; Esquivel et al., 2013), prior to assembly of the corresponding cell surface structures which are required for a set of physiologically relevant processes such as motility, adhesion, and biofilm generation (Tripepi et al., 2010; Pohlschroder and Esquivel, 2015). To the best of our knowledge, studies on the regulation of PibD expression have not been performed, however, in a quantitative proteomics study it was observed that the concentration of this protease was decreased and its electrophoretic mobility was affected in the *H. volcanii rhoII* KO mutant (MIG) in comparison to the parental strain (Costa et al., 2018) (see below). In addition, PibD substrates PilA1 and FlgA1 were also affected in the rhomboid mutant, as they evidenced altered electrophoretic mobility along with accumulation at the membrane (PilA1) and a decrease in concentration in the extracellular fraction, indicative of impaired archaellum assembly (Flga1). Whether these effects result from a direct action of RhoII on PibD and/or its substrates or, alternatively, are an indirect consequence remains to be clarified.

#### Processing and Glycosylation of the Cell Wall (S-Layer Glycoprotein)

The cell surface of haloarchaea is surrounded by a proteinaceous 2D structure, the S-layer, which in the case of *H. volcanii* is composed by a sole protein, the S-layer glycoprotein (SLG). Recent studies confirmed that the SLG is arranged in a hexagonal structure tightly associated with the cell membrane and stabilized by positively charged ions (von Kügelgen et al., 2021). The SLG not only provides protection to the cell but is important for its interaction with the environment and other cells (Albers and Meyer, 2011; Klingl, 2014; Sivabalasarma et al., 2021). In *H. volcanii* the SLG is matured through extensive post-translational modifications (PTMs) including proteolytic processing (Abdul Halim et al., 2013), lipidation (Konrad and Eichler, 2002a) and glycosylation (Konrad and Eichler, 2002b; Eichler et al., 2013; Kaminski et al., 2013; Kandiba and Eichler, 2014; Parente et al., 2014; Kandiba et al., 2016). This glycoprotein has been used as a reporter protein to study the N-glycosylation process in archaea. In *H. volcanii* two glycosylation pathways have been identified having two distinct sets of enzymes denoted as Agls (archaeal glycosylation) enzymes. The AglB-dependent pathway is responsible for the biosynthesis of a pentasaccharide (glucose–glucuronic acid–galacturonic acid–methyl-glucuronic acid–manose) at positions N13, N83, N274, and N279 of the SLG (Kandiba et al., 2016) also found decorating other glycoproteins (Esquivel et al., 2016; Schulze et al., 2021). The Agl15-dependent pathway mediates the assembly of a tetrasaccharide (sulfated hexose–hexose–hexose–rhamnose) originally identified at position N498 under at low salt conditions (Kaminski et al., 2013). A recent in-depth glycoproteomics analysis identified an additional *N*-glycosite at N370 harboring AglB-dependent glycans. Also, positions N274 and N279 were found to be modified with AglB and Agl15-dependent glycans in control salt conditions (18% NaCl), while AglB-dependent glycosylation was also observed at N498, suggesting that Agl15-dependent *N*-glycosylation is not dependent on low salt conditions and that both pathways can modify the same *N*-glycosites (Schulze et al., 2021). A PTM at position N732 with a sulfoquinovose-containing oligosaccharide was reported even though the pathway responsible for its assembly has not been addressed (Parente et al., 2014).

*Haloferax volcanii* encodes two rhomboid homologs, RhoI (HVO_1474) and RhoII (HVO_0727) (Table 1). KO mutants in the gene encoding *rhoII* have been constructed and characterized phenotypically and at the proteome level. *Haloferax volcanii* cells with a deletion in *rhoII* produced a truncated oligosaccharide bound at N732 in the SLG, evidencing the participation of rhomboids in the regulation of protein glycosylation in this haloarchaeon (Parente et al., 2014). This hypothesis was supported by the observation that several proteins of the *N*-glycosylation pathway were affected in abundance and/or electrophoretic mobility in the *H. volcanii* mutant compared to the parental strain (Costa et al., 2018). Among these, GalE epimerase/dehydratase (HVO_1576), Agl12 (HVO_2059), Agl18 (HVO_2060), an undecaprenyl pyrophosphate synthetase (HVO_2318), and phosphohexomutase (HVO_2989) are soluble proteins, meaning that the influence of RhoII on their differential processing/stability is most likely indirect. Conversely, the membrane-associated predicted flippase Agl15 (HVO_2055), which displayed differential electrophoretic mobility, is an integral membrane protein and could be a potential endogenous substrate of RhoII. Recently, it was reported that the rhomboid protease RHBDL4 controls protein glycosylation in the endoplasmic reticulum in humans by processing the oligosaccharyl-transferase subunits releasing the products to the cytoplasm for proteasome degradation (Knopf et al., 2020). Altogether, this evidence suggests that the participation of Rhomboids as regulators of the *N*-glycosylation process is a conserved trait.

The proteomic analysis of the LonB mutant HVLON3 (Cerletti et al., 2015, 2018) revealed that a number of glycosyltransferases (GTs) were affected in their abundance and/or turnover depending on the cellular concentration of LonB. Specifically, HVO_0704, HVO_1529 (AglG), and HVO_2049 (Agl10) were selected as potential LonB targets as their pattern of degradation was notoriously enhanced when the cellular LonB concentration was increased (Cerletti et al., 2018). A comparative analysis of the SLG glycosylation pattern in wt vs the LonB mutant will help to clarify the participation of LonB in the regulation of this process. Based on the available evidence, it can be hypothesized that the membrane proteases RhoII and LonB are likely regulatory factors of the (SLG) *N*-glycosylation process in *H. volcanii*.

In *H. volcanii* insertion of the SLG into the cell surface is achieved by a novel protein anchoring mechanism that involves the proteolytic processing of a C-terminal sorting signal by the peptidase archaeosortase A (ArtA) followed by lipid attachment (Abdul Halim et al., 2013). The sorting signal recognized by ArtA is a tripartite structure containing PGF, a hydrophobic fragment (TM) and a stretch of basic residues localized at the C-terminus or in other regions of the substrate protein (Abdul Halim et al., 2015, 2017). Site directed mutagenesis studies of *H. volcanii* ArtA showed that the highly conserved Cys^173^, Arg^214^, and Arg^253^ of ArtA are critical for its catalytic activity (Abdul Halim et al., 2018). Mutants lacking the *artA* gene (HVO_0915) evidence poor growth, differences in cell shape and were impaired in motility and conjugation (Abdul Halim et al., 2013, 2015, 2017). Recently, it was shown that HvPssA and HvPssD, homologs of the bacterial phosphatidyl serine synthase and phosphatidyl serine decarboxylase, respectively, are involved in the maturation of ArtA substrates. This conclusion was supported by the observation that mutants lacking these genes were impaired in the proteolytic processing and/or lipid attachment and showed similar phenotypes as those displayed by Δ*artA* mutants. Based on co-localization experiments, it was suggested that the interaction between HvPssA, HvPss, ArtA, and SLG occurs at the midcell during growth and cytokinesis and a model was proposed in which recruitment of ArtA, HvPssA, and HvPssD to the mid cell may promote the insertion of newly matured SLG to the cell wall at midcell contributing to cell division and elongation (Abdul-Halim et al., 2020).

### Expression and Potential Functions of Putative Membrane Protease Homologs

Based on proteomics studies performed in *H. volcanii*, various proteins related to membrane protease families were detected, including proteins related to CAAX prenyl proteases, Site 2 Proteases (S2P) and HtpX. Some of these showed differential expression depending on the environmental condition and/or in strains lacking other proteases (Rho II). This preliminary evidence suggests that these proteins (or at least some of them) may be functional enzymes in this organism.

Increased HtpX transcript levels under heat shock were detected in *Pyrococcus furiosus* (Shockley et al., 2003) as well as increased protein abundance of HtpX during oxidative stress in *H. volcanii* (McMillan et al., 2018) suggesting that, as in bacteria, HtpX protease likely participates in membrane protein quality control in archaea. Three HtpX homologs (HVO_0102, HVO_2904, and HVO_A0045) were detected in the *H. volcanii* proteome (Archaeal Proteome Project data base)^1^ (Schulze et al., 2020). Of these, HVO_A0045 showed differential expression (increased abundance) in a strain lacking the rhomboid homolog RhoII (Costa et al., 2018), suggesting some sort of stress in the membrane that may trigger the regulation of this putative HtpX protease.

Four S2P protease homologs with different structure and putative regulatory domains were detected in the *H. volcanii* proteome, HVO_0285 with a CBS domain (binds ATP, AMP or S-adenosyl derivatives), HVO_1870 with PDZ domain (recognizes the C-terminus of target protein) and two, HVO_1555 and HVO_1862, without an additional domain (Schulze et al., 2020). Elimination of the rhomboid protease gene encoding RhoII increased the protein abundance of HVO_1870 as well as that of the PrsW-like homolog (HVO_0408) (Costa et al., 2018). In *Bacillus subtilis* PrsW acts as S1P in the signaling cascade leading to degradation of an anti-sigma factor under stress conditions (Heinrich et al., 2009). Whether there is a functional connection between these proteins (PrsW/HVO_1870) in *H. volcanii* is an issue to be investigated in the future.

Homologs of the CAAX prenyl-protease family are present in all domains of life. In eukaryotes they participate in signaling pathways, however, their function is scarcely known in prokaryotes some members proposed in bacteriocin maturation (Pei and Grishin, 2001). The gene encoding a hypothetical protein related to the family of CAAX prenyl proteases (HVO_0784) is expressed as a transcription unit together with *lonB* mRNA in the haloarchaea *Natrialba magadii* and *H. volcanii* (Sastre et al., 2010; Cerletti et al., 2014). Deletion of this gene did not affect the viability and growth performance in *H. volcanii* cells but produced an impact on its proteome (Cerletti et al., 2015).

**FIGURE 1:**
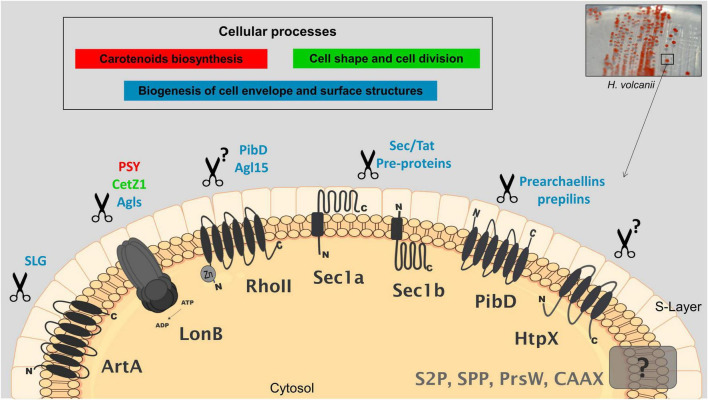
Cellular processes and potential targets of membrane-localized proteases in *Haloferax volcanii.* Proteases that have been characterized by genetic, biochemical and/or proteomics studies are shaded in black; those uncharacterized are shaded in gray; proposed biological functions and associated potential targets are indicated with the same color. Genetics combined with proteomics have shown that LonB affects the degradation of key proteins involved in carotenogenesis (PSY), cell shape determination (CetZ1) and likely the glycosylation of SLG (several GTs). Rhomboid RhoII is implicated in the biogenesis of a sulfoquinovose-containing oligosaccharide as a mutant deleted in the *rhoII* produces a truncated version of this oligosaccharide. PibD and Agl15 are suggested as potential targets of RhoII based on differential proteomics on wt and a mutant lacking *rhoII*. Anchoring of the SLG into the cell surface involves the proteolytic processing of a C-terminal sorting signal by the peptidase archaeosortase A (ArtA) followed by lipid attachment, this mechanism has been substantiated by various types of evidence. SPIs Sec11a/b and TFPP-like PibD process pre-proteins for membrane insertion, secretion and for cell surface structures. Protease homologs of HtpX, S2P, SPP, CAAX prenyl-proteases, and PrsW have been detected by differential proteomics. HtpX was suggested to participate in stress response. Additional details and the corresponding references are described in the text.

The current knowledge on the proposed biological functions and targets of membrane proteases in *H. volcanii* is depicted in Figure 1, which can be used as a working model for future research.

## Concluding Remarks

In spite of the fact that proteolysis plays a central role in many processes that occur within the context of the membrane and cell envelope of prokaryotes, the physiological relevance, endogenous targets and processes regulated by membrane-localized proteases are scarcely known in archaea. In the last years first insights on the processes controlled by the regulatory proteases LonB and Rhomboids and some potential physiological targets have begun to emerge as well as a better understanding of the mechanism of SLG maturation mediated by archaeosortase ArtA. The participation of membrane proteases in the control of key cellular processes such as carotenogenesis may guide biotechnological developments including overproducing strains as a source of bioactive compounds with novel properties.

Still, this is an area open to research and studies are encouraged to contribute to extend our fundamental knowledge on proteases, archaeal physiology as well as aid in the design of biotechnological applications of these “unusual” organisms and their biomolecules.

## Author Contributions

All authors made substantial contributions to the acquisition, analysis and interpretation of data for this review, critically reviewed and edited the manuscript, approved the final version before submission to publication, and agreed to be accountable for all aspects of the work in ensuring that questions related to the accuracy or integrity of any part of the work are appropriately investigated and resolved.

## Conflict of Interest

The authors declare that the research was conducted in the absence of any commercial or financial relationships that could be construed as a potential conflict of interest.

## Publisher’s Note

All claims expressed in this article are solely those of the authors and do not necessarily represent those of their affiliated organizations, or those of the publisher, the editors and the reviewers. Any product that may be evaluated in this article, or claim that may be made by its manufacturer, is not guaranteed or endorsed by the publisher.

## Footnotes

1
https://archaealproteomeproject.org/
